# Cytotype coexistence in the field cannot be explained by inter-cytotype hybridization alone: linking experiments and computer simulations in the sexual species *Pilosella echioides* (Asteraceae)

**DOI:** 10.1186/s12862-017-0934-y

**Published:** 2017-03-23

**Authors:** Jindřich Chrtek, Tomáš Herben, Radka Rosenbaumová, Zuzana Münzbergová, Zuzana Dočkalová, Jaroslav Zahradníček, Jana Krejčíková, Pavel Trávníček

**Affiliations:** 1 Institute of Botany, The Czech Academy of Sciences, CZ-252 43 Průhonice, Czech Republic; 2 Department of Botany, Faculty of Science, Charles University in Prague, CZ-128 01 Prague, Czech Republic; 3 Department of Botany, Natural History Museum, National Museum, CZ-193 00 Prague – Horní Počernice, Czech Republic; 4 Faculty of Agriculture, University of South Bohemia, CZ-370 05 České Budějovice, Czech Republic

**Keywords:** Polyploidy, Cytotype diversity, Mating interactions, *Pilosella echioides*, Minority cytotype exclusion, Triploid bridge

## Abstract

**Background:**

Processes driving ploidal diversity at the population level are virtually unknown. Their identification should use a combination of large-scale screening of ploidy levels in the field, pairwise crossing experiments and mathematical modelling linking these two types of data. We applied this approach to determine the drivers of frequencies of coexisting cytotypes in mixed-ploidy field populations of the fully sexual plant species *Pilosella echioides*. We examined fecundity and ploidal diversity in seeds from all possible pairwise crosses among 2x, 3x and 4x plants. Using these data, we simulated the dynamics of theoretical panmictic populations of individuals whose progeny structure is identical to that determined by the hybridization experiment.

**Results:**

The seed set differed significantly between the crossing treatments, being highest in crosses between diploids and tetraploids and lowest in triploid-triploid crosses. The number of progeny classes (with respect to embryo and endosperm ploidy) ranged from three in the 2x-2x cross to eleven in the 3x-3x cross. Our simulations demonstrate that, provided there is no difference in clonal growth and/or survival between cytotypes, it is a clear case of minority cytotype exclusion depending on the initial conditions with two stable states, neither of which corresponds to the ploidal structure in the field: (i) with prevalent diploids and lower proportions of other ploidies, and (ii) with prevalent tetraploids and 9% of hexaploids. By contrast, if clonal growth differs between cytotypes, minority cytotype exclusion occurs only if the role of sexual reproduction is high; otherwise differences in clonal growth are sufficient to maintain triploid prevalence (as observed in the field) independently of initial conditions.

**Conclusions:**

The projections of our model suggest that the ploidal structure observed in the field can only be reached via a relatively high capacity for clonal growth (and proportionally lower sexual reproduction) in all cytotypes combined with higher clonal growth in the prevailing cytotype (3x).

## Background

Polyploidy is an important part of the biology of angiosperms and a major force in their evolution [1–5]. It can broadly affect gene regulation and developmental processes that may cause shifts in morphology, breeding systems and ecological tolerance [1, 6, 7].

Species groups or single species with variation in ploidy level are suitable model systems for studies aimed at understanding recent polyplodization processes and their evolutionary importance (e.g. [8–12]). Different cytotypes may be completely separated geographically, or coexist in close proximity within a primary or secondary contact zone (e.g. [13, 14]), often intermingled within single populations [1, 12, 14–18].

The dynamics of mixed-ploidy populations are presumed to be dominated by minority cytotype exclusion, a frequency-dependent process in which a rare cytotype (as a rule diploid or tetraploid) experiences a transmission disadvantage due to the combined effect of a high proportion of inter-cytotype crosses and strong incompatibility between cytotypes (triploid block [19–21]). On the other hand, the coexistence of different cytotypes can be more or less stable thanks to either (i) reproductive isolation as a consequence of a diverse array of pre- and postzygotic inter-cytotype breeding barriers [22–26] or, alternatively, (ii) an absence of breeding barriers linked with interfertility between cytotypes [27, 28]. The most interesting are systems with free mating because they allow us to study intercytotype interactions and evolutionary dynamics of mixed-ploidy stands.

One factor which can facilitate the formation and establishment of polyploids is additional asexual reproduction by clonal growth. Two hypotheses attempting to explain the association between polyploidy and clonal growth have been proposed: (i) that clonal reproduction is a precondition for polyploid evolution, and (ii) that polyploidy enhances the incidence or degree of clonality [29–31]. While the former supposes that clonality could facilitate the establishment of polyploid populations by reducing reliance on immediately available mates, the latter points out direct or indirect (by altering natural selection) effects of genome duplication on clonal growth. However, there are only a few empirical tests of these predictions. The association between clonal reproduction and polyploidy was first reported in the late 1940s [29]. Recent studies aimed at the effect of ploidy variation on genotypic diversity in clonal species [32, 33], and more specifically on clonal size [34], however, indicate that polyploids are not more clonal compared to conspecific diploids and that it is hardly possible to draw any general conclusions about the interactions between clonality and polyploidy. On the other hand, it has been shown that lateral spread is a prerequisite for polyplodization, as it can compensate for the lower efficiency of generative reproduction in some polyploids.

The role of triploids (or odd ploidy levels in general) in mixed-ploidy populations remains a matter of debate. Nearly all models of polyploidy assume that triploids are either nonviable or have significantly lower fitness, which reflects the widely held view that diploids and their polyploid derivatives are isolated by postzygotic genetic barriers (e.g. [20, 35, 36]), referred to as the ‘triploid block’ [19, 21]. This assumption, however, is not fully supported by data on wild species with mixed-ploidy populations, e.g. on *Chamerion angustifolium* [27, 37, 38]. Larger proportions of triploids found in some populations of *Pilosella rhodopea* (up to 97%, but clonal growth is expected here [39]), *Chamerion angustifolium* (9%, [40]) and *Galax urceolata* (up to 27%, [8]) suggest that triploids may be more viable and fertile than is commonly presumed. Triploids can not only contribute to the formation of tetraploids via the triploid bridge but can also play a role both in the stable coexistence of diploids and polyploids and in the fixation of tetraploids [27]. A better understanding of the behaviour and evolutionary importance of triploids can substantially alter the view not only on mechanisms driving the dynamics of mixed-ploidy populations but also on the general issues of polyploidy evolution.

Processes potentially driving the dynamics of mixed-ploidy populations can be explored by a targeted approach. In principle, they are best explored by a combination of (i) detailed screening of cytotype frequencies in field populations, (ii) crossing experiments among all cytotypes to reveal the fecundity of maternal plants and ploidal diversity of the progeny from particular crosses, and (iii) computer simulations putting together the outcomes of crossing experiments and field data. To date, this approach has been only scarcely applied. One still outstanding exception is the study of the effect of triploids on tetraploid evolution in *Chamerion angustifolium* [27].

In this paper, we aim to explain the proportions of individual ploidies in a field mixed-ploidy population of *Pilosella echioides* (Asteraceae) by the proportion of ploidies they produce in inter-cytotype crosses (progeny analysis). We used mathematical models to examine whether specific values of their parameters (especially longevity and clonality) can explain discrepancies between field data and progeny analysis data. *Pilosella echioides* is a species with ploidal diversity (2x – 6x) and a high proportion (up to 70%) of triploids. In most of its geographic range (largely in steppe grasslands from southern Russia westwards to Central Europe [41]), *P. echioides* is a perennial, sexual and self-incompatible species with a capability for clonal growth *via* daughter rosettes. It possesses several features that make it a good candidate for such studies: (i) Up to five cytotypes may coexist within one population at a very fine spatial scale; (ii) It is an extremely dynamic system of cytotype coexistence due to the fertility of all cytotypes and interfertility amongst them, both in experiments and presumably also in wild populations; and (iii) No significant differences between cytotypes in morphology, phenology or habitat preferences were found [28].

We first examined the fecundity of maternal plants and ploidal diversity in seeds in a complete set of within- and between-ploidy crossing experiments among 2x, 3x and 4x plants. Secondly, we performed computer simulations of dynamics of theoretical populations of individuals whose progeny structure is identical to that determined by the hybridization experiment, examined over a broad range of initial conditions, including those found in the field. We used these simulations to ascertain the equilibrium proportions of individual cytotypes and to compare them with field data on the frequency of cytotypes (diploids – 6%, triplioids – 73%, tetraploids – 20% and pentaploids – 1% [28]). Finally, as the mating structure alone cannot account for the observed frequencies, we examine the possible impacts of two other parameters which may be responsible for the cytotype frequencies in the field, namely plant longevity and clonal growth. Due to identical morphology and niche preferences, shifts in the intensity of clonal growth may explain fitness differences between cytotypes. Thus, we incorporated this parameter into the model to examine their explanatory potential for the patterns found in the field.

## Methods

### Parental plants for crossing experiments

All plants used in our crossing experiments came from the locality of Havraníky, a heathland near the town of Znojmo, SW Moravia, Czech Republic (48°48' N, 15°59' E), where five cytotypes (2x–6x) of *Pilosella echioides* co-occur in mixed-ploidy populations ([28]; all data on the frequency and spatial pattern of cytotypes used in this paper were also adopted from this study). The population can be considered a closed system, although gene flow from neighbouring populations cannot be fully excluded. However, short-distance pollen transfer (mostly between neighbouring plants) strongly prevails in dense populations of insect-pollinated plants. Secondly, long-distance pollen transport plays a role in a long-term perspective whereas its effect on a short time scale is low. Seed dispersal by wind is reduced, as the patches of heathland are more or less separated by forests.

Young plants (rosettes) were collected in spring 2009, transferred to an unheated greenhouse in the Experimental Garden of the Institute of Botany, Průhonice (49°59'40"N, 14°34'01"E) and grown to flowering. The ploidy level of each plant was checked by flow cytometry (see below). Subsequently, 22 plant of each ploidy level (2x, 3x and 4x; higher ploidy levels are very rare in the field and were not included in our crossing design) were randomly chosen, 18 plants per ploidy level were randomly grouped into crossing blocks (see below), and four plants per ploidy were retained to potentially replace withered or damaged plants. Voucher specimens are preserved in the Herbarium PRA.

### Crossing design

Reciprocal crosses among plants of all ploidal combinations were carried out in the summer (June–July) of 2009, i.e. 2x × 2x, 3x × 3x, 4x × 4x, 2x × 3x, 2x × 4x, 3x × 2x, 3x × 4x, 4x × 2x and 4x × 3x (total of 9 blocks: reciprocal heteroploid crosses – e.g., 2x × 4x and 4x × 2x – were conducted in one block each, i.e. 6 crossing blocks + 3 homoploid blocks). In homoploid crosses, six plants per block were crossed; in heteroploid crosses, six plants per each ploidy were crossed (i.e. 12 plants per block). At least four different pollen donors were mated with every maternal plant, each at least with two capitula. Each maternal plant capitulum was pollinated by only a single pollen donor. Inflorescences were isolated in nylon bags before anthesis and between pollinations. The crosses were carried out in the stage of stigma receptivity (when bifurcate stigmas protrude from the flowers) by rubbing the whole parental capitula together repeatedly on three consecutive days. Selected capitula in most of the maternal plants were bagged and left untreated (to detect the capacity for autonomous selfing) or pollinated by another capitulum from the same plant to detect the self-incompatibility (previously conducted experiments revealed all cytotypes of *P. echioides* to be self-incompatible at our locality as well as in neighbouring regions [42]).

### Maternal plant fecundity and ploidal diversity in developed seeds

Seeds from each capitulum were collected and kept separately. The number of full (developed, black) cypselae (referred to as “seeds” for simplicity) was counted in each capitulum and expressed as a proportion of the total number of flowers in a capitulum (seed set). The proportion of full seeds among blocks was compared using a one-way ANOVA and Scheffé’s post-hoc test. The influence of interactions among ploidy levels, parental combination and direction of cross on the seed set was tested using GLM factorial ANOVA. Data were arcsine transformed. Full seeds from each parental combination (capitula of the maternal plant pollinated with the same male parent) were put together and randomly separated into two equal batches except for seed sets from 3x × 3x crosses (due to a very low number of full seeds). The first half and all seeds from 3x × 3x crosses were analysed using the Flow Cytometric Seed Screen method (FCSS [43]; not all seeds from 2x × 2x and 4x × 4x crosses were analysed because of the high number of seeds and relative homogeneity with respect to ploidy levels). For the second half, germination frequencies in the growth chamber and the common garden were estimated.

### Flow cytometric seed screen (FCSS)

**Fig. 1:**
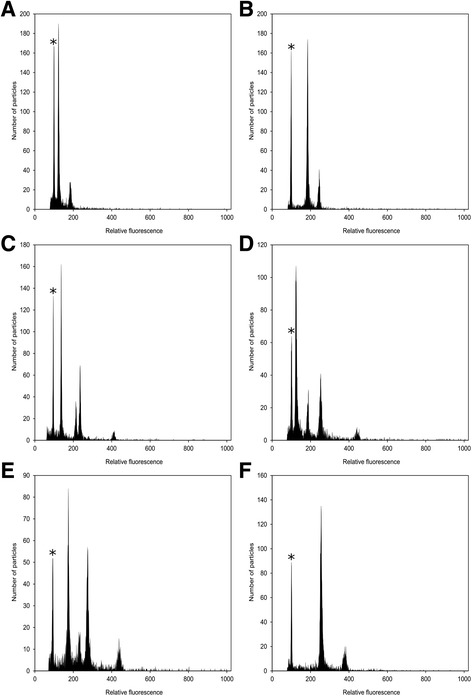
Resulting histograms of FCSS analyses of cypselae from different inter-cytotype crossings. **a** Heteroploid crossing of 2x × 3x which typically provides 2x embryo and 3x endosperm. There are two alternative explanations – selfing of diploid maternal plant or more likely fusion of reduced gametes of both parents; **b** Heteroploid crossing of 2x × 4x with typical 3x embryo and 4x endosperm pointing to fusion of two reduced gametes of both cytotypes; **c** Heteroploid crossing of 3x × 2x with aneuploid seed derived from diploid and euploid seed with 4x embryo and 7x endosperm. Typical behaviour of maternal triploid plant producing either aneuploid gametes or fully unreduced gametes resulting in higher ploidy level of progeny; **d** Homoploid crossing of 3x × 3x resulting to euploid progeny – 2x embryo and corresponding 3x endosperm from fusion of two fully reduced gametes and 4x embryo and 7x endosperm from fusion of unreduced maternal and fully reduced paternal gametes; **e** Heteroploid crossing of 3x × 4x resulting in (i) euploid seed with 3x embryo and 4x endosperm pointing to fact that even maternal triploid plant can produce fully reduced gametes and (ii) aneuploid seed derived from pentaploid, documenting meiosis problem in 3x mother plant; **f** Typical output of seed screening from homoploid crossing of tetraploids resulting in 4x embryo and 6x endosperm

Direct flow cytometry on seeds was carried out to estimate the ploidy levels of embryos and endosperm. Our study revealed a high accuracy of the two-step procedure originally described by [44] and modified for plant applications [45]. Usually two mature seeds (with pericarp and pappus) were chopped with an appropriate amount of an internal standard (*Bellis perennis*; 2C = 2.96 pg according to [46]) in a Petri dish containing 0.5 ml of ice-cold Otto I buffer (0.1 M citric acid, 0.5% Tween 20). The suspension was filtered through a nylon mesh (loop size 42 μm) into 3.5 ml cuvettes and incubated for five minutes at room temperature. Isolated nuclei were stained using 1 ml of Otto II buffer (0.4 M Na_2_HPO_4_.12H_2_O) supplemented with the AT-selective fluorochrome DAPI (4′,6-diamidino-2-phenylindole) and β-mercaptoethanol at final concentrations of 4 μg · ml^−1^ and 2 μl · ml^−1^, respectively. After five-minute incubation at room temperature, the relative fluorescence of 3000 particles was recorded with a PA-II cytometer (Partec GmbH, Münster, Germany) equipped with a mercury lamp as the source of excitation light in the UV spectrum (ca 420 nm). The resulting histograms were analysed in FloMax software (Partec GmbH), and both fluorescence intensities of embryo and endosperm peaks were recorded to evaluate the ploidy level of parental gametes involved in the origin of each particular seed. Because one or two seeds were used in each analysis, 2–4 peaks were recognized in resulting histograms. Prior knowledge of parental combination of each seed allowed us to assign corresponding embryos and endosperm together and to determine their ploidy level precisely (Table 1, Fig. 1). For distinguishing between eu- and aneuploids, we used a simple rule inferred from known ratios of fluorescence intensities of euploids and the internal standard [28]. To regard a particular plant (embryo in a seed) as euploid, the deviation from mean fluorescence intensities for a given ploidy was not allowed to be greater than 5%. Hence, all plants/embryos whose fluorescence intensity deviated from the closest ploidy level by less than this threshold were assessed as euploids. Any plant that did not fulfil this criterion was considered aneuploid. Mean fluorescence intensities for particular ploidy level with their allowed deviations were used as follows: diploid – 1.25 (1.19–1.31), triploid – 1.90 (1.81–1.99), tetraploid – 2.49 (2.37–2.61), pentaploid – 3.18 (3.02–3.34) and hexaploid – 3.67 (3.49–3.85) (adopted from [28]).

**Table 1:** Proportion of full seeds (mean ± SD) in capitulum in experimental crosses

	Paternal parent		
Maternal parent	2x	3x	4x
2x	0.52^ef^ ± 0.28 (41; 2525)	0.23^abcd^ ± 0.23 (31; 1692)	0.66^f^ ± 0.13 (39; 2343)
3x	0.22^abc^ ± 0.16 (38; 2007)	0.06^a^ ± 0.06 (86; 4280)	0.30^bcd^ ± 0.15 (61; 2750)
4x	0.44^de^ ± 0.17 (44; 2729)	0.15^ab^ ± 0.1 (54; 2744)	0.40^cde^ ± 0.28 (46; 2955)

Means not sharing the same superscript were significantly different as determined by a one-way ANOVA and Scheffé's *post-hoc* test. The number of capitula and the total number of seeds (full + empty) are in parentheses

### The model

We used a simple algebraic model to project the outcomes of the crossing experiments against the equilibrium population structure in the field. We represented the results of the crossing experiments using a three-dimensional array, *M*_*ijk*_, whose elements give the probability that a cross between a maternal plant of the ploidy *i* and a paternal plant of the ploidy *j* will give rise to a daughter plant with ploidy *k*. Indices *i*, *j* and *k* run from 2 to the maximum known ploidy of the system [7]. Elements of this array are standardized so that ∑_*k*_ *M*_ijk_ = 1 for all *i* and *j*. Further, we define relative fertility of each cross, *f*_*ij*_, which contains relative numbers of seeds resulting from each cross. For the sake of simplicity, we define *f*_22_ = 1 and express other fertilities relative to it.

In the simplest model (further referred to as the Simple Model) we assume that the plants are monocarpic, have non-overlapping generations and reproduce only sexually. We also assume that population sizes are sufficiently large for demographic stochasticity to be negligible because ploidal structure in the field has been estimated based on 2388 plant individuals [28], and total population size can be roughly estimated to be on the order of 10^4^. Assuming random pollen transfer among all individual irrespective of their ploidy level, the dynamics of such a system can be modelled using a set of simple recurrent equations where the proportion of plants of ploidy *k* at time *t* + 1 is determined by the proportions of plants in all ploidy levels at time *t*, and fertility and crossing data:

1 $$ {P}_k\left( t+1\right)={\displaystyle {\sum}_{i, j}{f}_{i j}{M}_{i j k}{P}_i( t){P}_j( t)} $$

where *P*_*i*_(*t*) denotes proportion of plants in ploidy *i* at time *t*. As we have no data on actual population dynamics and are interested only in proportions of individual ploidies, these proportions are further normalized at each step so that ∑_*k*_ *P*_*k*_(*t* + 1) = 1. This normalization removes all possible effects of changes in absolute population sizes, including year-to-year environmental variation because there is no indication that such changes would affect individual ploidies differently. To obtain population projections, the Eq. (1) was iterated both to see the transient behaviour due to different sets of initial conditions and to obtain equilibrium ploidal proportions. The equilibrium was assumed when relative changes in ploidal proportions were smaller than 10^−6^; this was typically attained after 100 steps. In this model, time steps are best viewed as generations (i.e. time needed for a seedling to reach the reproductive stage). We use this model as a null model for a system where ploidies do not differ in their vegetative growth parameters and the system is driven by sexual reproduction only.

In an alternative model, we assume that plants are capable of reproducing clonally and are not monocarpic (Clonal Model), the proportion of plants of ploidy *i* at time *t* is the sum of relative contributions of sexual reproduction and clonal reproduction (including survival of already fertile plants; plants are assumed to be polycarpic). The proportion of plants of ploidy *k* at time *t* + 1 can then be expressed as follows:

2 $$ {P}_k\left( t+1\right)=\left(1- g\right){c}_k{P}_k( t)+ g{\displaystyle {\sum}_{i, j}}{f}_{i j}{M}_{i j k}{P}_i( t){P}_j( t) $$

where *g* is the overall relative contribution of the sexual process to the population structure and *c*_*k*_ is the ploidy-specific capacity for clonal growth and/or survival for ploidy level *k*. It should be noted here that the quantity 1 - *g* expresses the contribution of both species longevity and clonal growth to the population structure. The proportions were again normalized at each step. In the general case, we assume that the capacity for clonal growth may differ between individual ploidies. In this model, time steps should be defined as years (or other time units independent of the plant life cycle).

### Model parameterization

The elements of the array *M*_*ijk*_ and relative fertilities of individual interploidal crosses (*f*_*ij*_) were known from the crossing experiments for *i*, *j* = 2, 3, 4. We had no data for crosses that involved pentaploids or higher ploidy levels, though such plants did arise from the crossing experiments and were found in the field (pentaploids and rarely hexaploids). To ensure a closed system, we assumed that fertilities of crosses involving ploidy levels higher than tetraploids were zero. Experiments with non-zero realistic values of penta- and hexaploid fertilities and *M*_*ijk*_ elements yielded very similar values as the variant with zero values. These assumptions suffice for complete parameterization of the Simple model.

Additional assumptions are necessary for the Clonal model because we have no information on the values of the parameters *g* and *c*_*k*_. Parameter *g* depends on the survival rate of already reproducing plants, the number of daughter ramets they bear each year, and on the establishment probability and time to maturity of plants from seeds. As we have only anecdotal information on these values, we assumed that this parameter may range from values close to zero (if clonal reproduction and survival largely prevail over seed reproduction) to one (if plants are monocarpic and nonclonal; then Eq. 2 becomes Eq. 1).

Parameter *c*_*k*_ expresses the relative capacity for clonal growth (and/or survival) of individual ploidy levels. Qualitative observation of clonal growth in pots in the experimental garden shows that diploid plants often produce fewer daughter ramets than triploids and tetraploids. This is also supported by different spatial pattern of diploid relative to triploid and tetraploid plants in the field (more clustered spatial pattern in tri- and tetraploids [28]). No such difference seems to exist between triploid and tetraploid plants. In the model, we assumed that clonal growth of diploids, triploids and tetraploids are independent of each other, but clonal growth of higher ploidies always equals that of tetraploids. We used tetraploids as a reference point also for diploids and triploids (i.e. *c*_*4*_ = 1) and examined relative clonal growth of these two ploidies in the range from 0.5 to 1.5.

Predictions of the model were obtained using numerical simulations based on parameter values and initial conditions (initial cytotype proportions). In all parameter settings, a wide range of initial conditions of proportions of diploid, triploids and tetraploids was examined, ranging from proportion of tetraploid of 0.01 to proportion of diploids of 0.01. In some simulations, we specifically examined the proportions found in the field as initial conditions.

## Results

### Seed set and ploidal diversity

Nearly all pollinated capitula yielded seeds, but the seed set differed significantly among the crossing treatments (F = 32.69, d.f. = 8, residual d.f. = 430, P < 0.001, Table 1). The highest seed set resulted from 2x × 4x and 2x × 2x crosses; 3x × 2x and 3x × 4x crosses gave rise to an intermediate proportion of seed set. The mean seed set in 3x × 3x was significantly lower than in all other crosses. The ploidy of the mother plant has a significant effect on the seed set (F = 49.60, d.f. = 2, P < 0.001), as did the ploidy of the male parent (F = 65.34, d.f. = 2, P < 0.001) and for the combined effect of both parents (male × female interaction) (F = 3.70, d.f. = 4, P < 0.006). All seeds from the control treatments (capitula bagged during flowering) were empty, indicating the absence of autonomous selfing, but induced selfing (mentor effect) could not be excluded based on our data.

Progeny classes in seeds with respect to both embryo and endosperm ploidy are provided in Table 2. The number of embryo (“Em” used below) + endosperm (“En”) progeny classes (incl. aneuploids as a separate class) ranged from three in 2x × 2x crosses to 11 in 3x × 3x crosses, generally being higher in crosses with the participation of triploid plants. All crosses with a triploid maternal parent yielded a diverse set of classes with a relatively high number of aneuploids (Table 2). While one progeny class strongly prevailed in most crosses (2 Em + 3 En in 2x × 2x and 2x × 3x, 3 Em + 4 En in 2x × 4x, 4 Em + 7 En in 3x × 2x, 3 Em + 5 En in 4x × 2x and 4 Em + 6 En in 4x × 4x), two classes with higher frequency were found in 3x × 3x (4 Em + 7 En and aneuploids), 3x × 4x (5 Em + 8 En and aneuploids) and 4x × 3x (3 Em + 5 En and 4 Em + 6 En) crosses. Diploid seeds were produced in five crossing blocks, but with higher frequency in 2x × 2x and 2x × 3x only; triploid seeds in 8 blocks, highest frequencies being in reciprocal 2x × 4x crosses, lower but still important in 4x × 3x crosses; tetraploid seeds in 8 blocks, highest frequency being in 4x × 4x crosses, lower but still important in 3x × 2x, 3x × 3x and 4x × 3x crosses (Table 2). Induced selfing (mentor effects) was very rare; it was observed with certainty only in the 2x × 4x crosses, and the frequency of selfed diploid seeds was very low (0.8%).

**Table 2:** Theoretical and observed (marked in bold) ploidy levels in seeds from experimental crosses

		Ploidy of male gamete											
		1x	2x	3x	4x	1x	2x	3x	4x	1x	2x	3x	4x
		2 x 2				3 x 2				4 x 2			
Ploidy of female gamete	1x	**2Em : 3En** (0.965)	**3Em : 4En** (0.002)	-	-	**2Em : 3En** (0.076)	**3Em : 4En** (0.007)	-	-	-	-	-	-
	2x	3Em : 5En	4Em : 6En	-	-	**3Em : 5En** (0.015)	4Em : 6En	-	-	**3Em : 5En** (0.852)	**4Em : 6En** (0.033)	-	-
	3x	-	-	-	-	**4Em : 7En** (0.694)	**5Em : 8En** (0.004)	-	-	-	-	-	-
	4x	-	-	-	-	-	-	-	-	**5Em : 9En** (0.006)	6Em : 10En	-	-
		Further classes: A - 0.033				Further classes: 4Em : 8En - 0.011, 4Em : 9En - 0.007, 4Em : 10En - 0.011, A - 0.175				Further classes: 3Em + 6En - 0.002, A - 0.107			
		2 x 3				3 x 3				4 x 3			
	1x	**2Em : 3En (0.964)**	**3Em : 4En (0.016)**	**4Em : 5En (0.004)**	-	**2Em : 3En (0.037)**	**3Em : 4En (0.01)**	**4Em : 5En (0.003)**	-	-	-	-	-
	2x	3Em : 5En	4Em : 6En	5Em : 7En	-	**3Em : 5En (0.003)**	4Em : 6En	**5Em : 7En (0.017)**	-	**3Em : 5En (0.432)**	**4Em : 6En (0.348)**	**5Em : 7En (0.022)**	-
	3x	-	-	-	-	**4Em : 7En (0.472)**	**5Em : 8En (0.058)**	**6Em : 9En (0.003)**	-	-	-	-	-
	4x	-	-	-	-	-	-	-	-	**5Em : 9En (0.048)**	**6Em : 10En (0.062)**	**7Em : 11En (0.004)**	-
		Further classes: A - 0.016				Further classes: 4Em : 9En - 0.01, 4Em : 10En - 0.003, A - 0.384				Further classes: A - 0.084			
		2 x 4				3 x 4				4 x 4			
	1x	-	**3Em : 4En (0.871)**	-	5Em : 6En	-	**3Em : 4En (0.095)**	-	5Em : 6En	-	-	-	-
	2x	-	**4Em : 6En (0.001)**	-	6Em : 8En	-	**4Em : 6En (0.005)**	-	6Em : 8En	-	**4Em : 6En (0.87)**	-	6Em : 8En
	3x	-	-	-	-	-	**5Em : 8En (0.322)**	-	7Em : 10En	-	-	-	-
	4x	-	-	-	-	-		-	-	-	**6Em : 10En (0.08)**	-	8Em : 12En
		Further classes: 2Em : 3En - 0.008, A - 0.12				Further classes: 3Em + 5En - 0.007, 3Em + 6En - 0.002, 5Em : 10En - 0.002, 5Em : 11En - 0.007, A - 0.56				Further classes: 4Em : 8En - 0.01, A - 0.05			

Ploidy levels of embryo (Em) and endosperm (En) in particular inter-cytotype experimental crosses of *Pilosella echioides* are provided. Frequencies of particular classes are given below. Further observed classes are given below each block. A – aneuploids

The frequency of reduced/unreduced male and female gametes that participated in particular crosses (as deduced from embryo and endosperm ploidy levels) is given in Table 2. Reduced gametes strongly prevail in both diploid and tetraploid parental plants. Female gametes of triploids proved to be mostly unreduced (3n), less frequently reduced to 2n and only very rarely reduced to n. Male gametes, by contrast, were mostly reduced to n. This fact indicates that triploid plants as paternal parents behave entirely different than in the maternal role.

### Model predictions

**Fig. 2:**
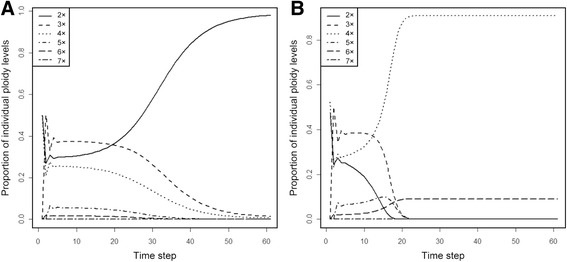
Examples of predicted time course of proportions of individual ploidy levels. Plants are assumed to be monocarpic with no clonal growth (Simple Model) and are parameterized with the data from the crossing experiment. **a** The initial proportion of diploids:tetraploids 0.5:0.5. **b** The initial proportion of diploids:tetraploids 0.45:0.55. These proportions have been selected to illustrate that small changes in initial proportions are predicted to lead to very different outcomes. For the full mapping of the space of initial conditions, see Fig. 3

**Fig. 3:**
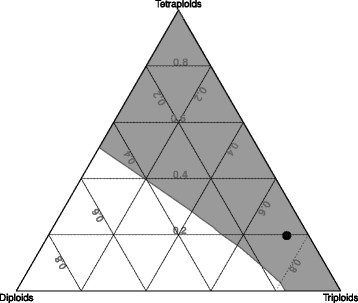
Final cytotype structure as a function of initial proportions of individual cytotypes. Different shading indicates regions in the space of the initial proportion of diploids, triploids and tetraploids that end in the stable state I (*white* region) and the stable state II (*grey* region) in the Simple Model parameterized by the field data. The triangular representation is used as proportion of diploids, triploids and tetraploids sum to one. The whole region of initial values was mapped using a step of 0.02; each combination of initial values was run for 200 steps (generations) to determine which stable state (Table 3) it is approaching. The *black dot* indicates the approximate position of the field population

**Fig. 4:**
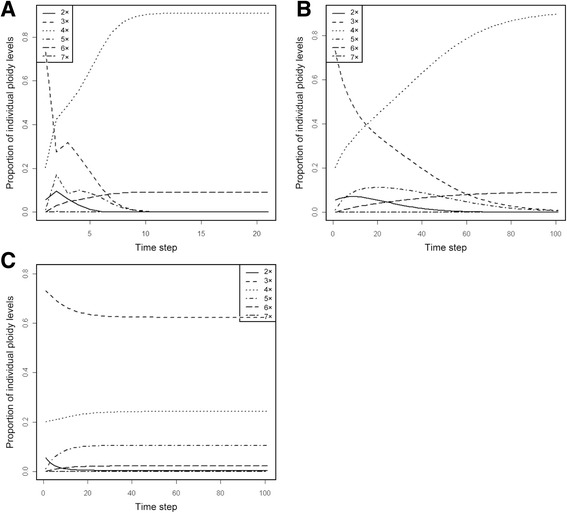
Predicted time course of proportions of individual ploidy levels starting with proportions found at locality. **a** plants are monocarpic with no clonal growth (Simple Model); **b** plants are perennial and clonal, clonal growth of all ploidy levels is identical (Clonal Model, *g* = 0.1); **c** plants are perennial and clonal, clonal growth of triploids is 1.2 times that of tetraploids and of diploids 0.8 times that of tetraploids (Clonal Model, *g* = 0.1, *c*_*2*_ = 0.8, *c*_*3*_ = 1.2). Note different scaling of the x axis. Hybridization data in all models are parameterized by the experiment

The Simple Model provides a deterministic, stable ploidal structure that depends only on the initial conditions and shows a clear case of minority cytotype exclusion. It has two stable states: (i) with prevalent diploids and lower proportions of other ploidies up to heptaploids, and (ii) with prevalent tetraploids, 9% of hexaploids and all other ploidy levels missing (see Table 3 and Fig. 2). None of these proportions is close to the proportions found in the field (see Table 3). If the system is composed initially only of diploids and tetraploids, diploids will prevail (i.e. the system will reach the first stable state) if their initial proportion is higher than 0.4972; otherwise tetraploids will prevail (i.e. the system will reach the second stable state). If triploids are present initially, they shift the balance towards the second stable state (Fig. 3). If the system is composed initially only of diploids and tetraploids, triploids arise abundantly during the early transient stages of the simulation, but their proportion never exceeds 50% (see Fig. 2 for an example). As a result, this model predicts that a system with the same proportions of ploidy levels as those found in the field at the Havraníky locality will converge rather fast to the second stable state, i.e. diploids, triploids and pentaploids will cease to be dominant over two generations and eventually disappear as the system reaches the stable state with only tetraploids and hexaploids present (over ca. 10 generations; see Fig. 4a).

**Table 3:** Two stable states in the Simple Model and the field-observed proportions of individual cytotypes

Ploidy level	2x	3x	4x	5x	6x	7x
Stable state I	0.9872	0.0092	0.0035	5.39E-05	1.88E-06	9.18E-08
Stable state II	0.0000	0.0000	0.9093	0.0000	0.0907	0.0000
Field-observed proportions	0.06	0.73	0.2	0.01	0	0

System of monocarpic plants with no clonal growth. Numbers in the first two lines in table are equilibrium proportions (after 500 iterations) of individual ploidy levels. If the system is initialized by diploids and tetraploids only, stable state I is reached when initial proportion of diploids is greater than 0.497, stable state II when initial proportion of diploids is smaller than 0.497

Introducing perennial plants and overlapping generations (*g* < 1; Clonal Model) yields a much larger variety of behaviours. If plants are long-lived and/or clonal but individual ploidy levels do not differ in this regard (*c*_*k*_ = 1 for all *k*), the system has the same stable state structure as the Simple Model; the only difference being the much slower dynamics of the system (Fig. 4b). For example, a system initialized with the same proportion of ploidies as those found in the field would retain triploid dominance over other ploidies for over 4 years if *g* = 0.4, over 14 years if *g* = 0.1, and over 144 years if *g* = 0.01. The relationship is asymptotic; for obvious reasons, triploids will remain dominant in the population if no generative reproduction takes place at all (*g* = 0). In such clonal model initialized by mixing diploids and tetraploids, the proportion of triploids never exceeds 0.5.

**Fig. 5:**
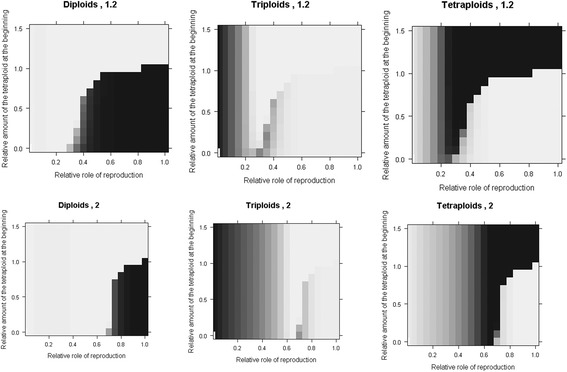
Role of seed reproduction and the initial proportion of tetraploids in the Clonal Model. Effects of the relative role of seed reproduction (*c*_*i*_) and the initial proportion of tetraploids on the final proportion of individual cytotypes in the Clonal Model. Each projection was run for 200 years beginning with only diploids and tetraploids with no other ploidy present. Shades of grey indicate the final proportion of the given ploidy from *white* (absent) to *black* (100%). *Upper row*: *c*_3_ = 1.2, *lower row*: *g c*_3_ = 2. The rate of clonal growth of all other cytotypes is one. *Left column*: diploids, *middle column*: triploids, *right column*: tetraploids. Hybridization data in the models are taken from the experiment

By contrast, differences between individual ploidies in their longevity and/or capacity for clonal growth do alter the equilibrium structure of the system (Fig. 4c). The intensity of this effect depends on the overall role of these processes relative to generative reproduction (i.e. the parameter *g*). If the role of longevity and/or clonal growth is weak (i.e. *g* is high, Fig. 5, right part of the plots), the final proportions of ploidies are largely determined by sexual process and show the pattern due to the minority cytotype exclusion that depends on the initial conditions in a frequency-dependent manner (Fig. 5). In such a system clonal growth of triploids cannot offset the disadvantage of triploids in sexual reproduction. But if clonal growth is important (i.e. *g* is below ca. 0.35; the exact position of the threshold depends on the relative clonal growth of triploids), the dynamics of the whole system change, and the initial conditions cease to matter (Fig. 5, left part of each plot). In such systems, higher relative clonal growth of triploids can cause triploids to prevail indefinitely. Their proportion is determined by the parameter *c*_*k*_, i.e. relative capacity for clonal growth (and/or survival) of individual ploidy levels (Fig. 5). Apparently, there are parameter combinations that predict a large and stable proportion of triploids matching those found in the field system (e.g. *g* < 0.1 and *c* > 1.5; see Fig. 4). Triploids in these systems are accompanied by tetraploids and pentaploids (which are constantly generated by these triploids).

## Discussion

### Offspring diversity

All cross treatments had more than one ploidy in their progeny, which suggests considerable variation in the frequency and quality of reduced and unreduced gametes (see also [47]). When the maternal parent was triploid, the crosses always yielded a high ploidal diversity in the progeny. While most of the embryo-endosperm progeny classes can be more or less easily explained by a combination of reduced/unreduced male and female gametes, the origin of certain other combinations is rather puzzling (Table 2, below particular tables). Another pending issue is the proportion and role of induced selfing caused by the occurrence of foreign pollen grains on the stigma (mentor effect). Selfing was observed with certainty only in the 2x × 4x crosses, and the frequency of selfed diploid seeds was very low (0.8%). In several other crosses, the proportion of induced autogamy could not be easily ascertained, e.g. 2 Em + 3 En seeds in the 2x × 3x crosses could have been a result of both the mentor effect and sexual fusion of haploid gametes of a diploid maternal and a triploid pollen parent. Induced autogamy can also be the case in 4 Em + 6 En in 4x × 2x and 4x × 3x crosses, and in some progeny classes in crosses with triploids as maternal parents. We tend to prefer the explanation invoking allogamous sexual fusion – in the case of the 2x × 3x cross, the contribution of reduced (n) male gametes from the triploid pollen parent because a similar pathway (n gametes from triploid pollen parent) was reliably proved in e. g. 3 Em + 5 En of the 4x × 3x crossing. Also is worth to mention the surprisingly high proportion of aneuploid seeds in some of the crosses, especially with triploids as female parents.

Relatively high frequency of unreduced female gametes in crosses with triploids as mother plants might evoke a participation of (most likely, as detected in the genus *Pilosella*) aposporous embryo sac replacing a sexual one, which can be taken as the first principal component of apomixis. The second component, parthenogenetic development of embryo, was not observed, both reduced and unreduced female gametes fused with male gametes and “complete” apomixis was also excluded in a series of castration experiments. A reproductive behaviour of facultatively apomicts in *Pilosella* considerably differ from that in our plants – as a rule, they produce a nearly complete seed set (versus low fertility of our plants), with usually prevaling apomictic seeds (sometimes incl. polyhaploids) and rather small portion of seeds originated from sexual fusion of reduced/unreduced female gametes and male gametes (versus no “complete” apomixis in our plants). Furthermore, apomixis as a heritable trait should be transferred to next generations, which also seems to be in a contradiction with our data, as no signs of apomixis have been detected in tetraploids.

### Comparison of predicted ploidal structure with field data

Ploidal proportions in seeds from experimental crosses and predictions from computer simulations are distinctly incongruent with the ploidal structure in wild populations of *Pilosella echioides* at the locality Havraníky [28]. While our simple model predicts two stable states (diploids prevailing, with lower proportions of other ploidies up to heptaploids, and tetraploids prevailing, with 9% hexaploids and all other ploidy levels missing), neither diploids nor tetraploids prevail in the field (5.6% diploids, 73.1% triploids, 20.2% tetraploids, 1.1% pentaploids, 0.04% hexaploids [28]). The same holds for the clonal model with equal capacity for clonal growth/longevity in all cytotypes. Even when the system is initialized by mixing diploids and tetraploids (mimicking contact between two separate ploidy levels due to e.g. migration), the proportion of triploids (prevalent in the field, 73.1%) in any transient state of the clonal model can never exceed 50%. This shows that progeny structure determined by the hybridization experiment cannot explain per se the pattern of ploidy in the field, either in equilibrium or not. These discrepancies can be explained by the clonal model (if there are inter-cytotype differences in clonal growth) or by other factors not considered in either of our models, namely differences in *in situ* germination rates, mortality of seedlings, seed dispersal and competitive ability, mating preferences and assortative mating caused by non-random distribution of cytotypes in the field.

The only possible scenario explaining the ploidal structure in the field within the framework of our model is a combination of either high longevity or strong clonal growth in all cytotypes, with a relatively higher rate in triploids. Differential longevity/clonal growth has a nonlinear effect on the behaviour of the system. At low but different longevity or clonal growth, the basic scenario of the minority cytotype exclusion (and, consequently, the two stable states) does not change. Above a certain limit, however, the overall dynamics of the systems suddenly shift, producing a system in which all cytotypes coexist in one stable state. This finding is rather general and qualitatively does not change with particular numerical values of longevity/clonal growth parameters. Although empirical data about their intensity in particular ploidy levels are not available, field observations in wild populations have revealed different spatial patterns in diploids and polyploids [28]. While polyploids often form more or less dense clusters, diploid plants are frequently more or less isolated from each other, indicating differential clonal growth across ploidy levels. *Pilosella echioides* lacks both above- and below-ground stolons (common in other *Pilosella* species), so clonal growth can only be realized *via* daughter rosettes. Moreover, fitness and the role of clonal growth in *P. echioides* may notably differ from other groups, as plants of all cytotypes have been proved to be self-incompatible [42]. In other groups, polyploids are often self-compatible (or at least do not suffer from selfing as much as diploids [48, 49]) and can benefit from within-clone (geitonogamous) selfing by reducing detrimental inter-cytotype hybridization. Generally, the relationship between polyploidy and clonality still remains an open issue, as recent studies provide partly contradictory results [31, 33, 34]).

In addition, alternative explanations such as higher *in situ* germination rates of triploid seeds, lower mortality of triploid seedlings or higher competitive ability of triploids in comparison with those of other cytotypes cannot be ruled out either. However, germination rates of triploid seeds in a growth chamber (mean 95.55%) are not significantly different from those in diploid seeds (94.4%); germination rates of tetraploid seeds are lower (85.3%) (Trávníček et al.). Secondly, although not revealed in experimental crosses using a pollen mixture [42], pollen preferences (with respect to ploidy level and mate choice) in multiple pollinations might also play a role in field populations (e.g. [50]). Prezygotic isolation mechanisms commonly reported from other species, such as flowering time divergence, pollinator preferences and foraging strategies or self-fertilization, most likely do not act in *P. echioides* (no significant differences between cytotypes in morphology and flowering phenology have been found; autogamy has not been confirmed). On the other hand, assortative mating can contribute to discrepancies between model predictions and field frequencies, as the distribution of cytotypes is not random and individual cytotypes often show a clustered spatial pattern. It is, however, unlikely that non-random mating *per se* could be responsible for the existing ploidal structure in the field. This would happen only if pollen transfer was strongly (at least several-fold) biased in favour of 2x- > 4x and 4x- > 2x pairs, namely at the expense of homoploid transfer. While this is theoretically possible, there is no evidence from this or any other plant system that such strong bias occurs.

Triploid seeds were detected in higher frequencies only in crosses with the participation of a diploid parent, namely 2x × 4x and 4x × 2x, which is also in striking contrast with the fact that diploids are fairly rare in the field. A distinctly lower, but still important, frequency of triploid seeds was observed in 4x × 3x crosses, which can at least partly explain the preferential coexistence of tri- and tetraploids in some patches. Crosses between triploid plants yielded a diverse seed set, albeit with an extremely low (1.3%) frequency of triploid seeds; thus, triploids can hardly be stabilized by homoploid crosses.

Another challenging issue, closely connected with the prevalence of triploids in the field population, is the frequency of pentaploids. In the models, the frequency of pentaploids increases with the frequency of triploids, irrespective whether it rises due to higher clonal growth or due to the system being in a transient state (e.g. because of contact between diploid and tetraploid plants/populations). On the contrary, the observed frequency of pentaploids in wild populations with predominating triploids is extremely low (1.1%, total of 2400 plants examined [28]). The absence of pentaploids in the field can be explained by lower fitness of their seedlings and young plants or lower germination rates, but direct evidence is lacking.

Analysis of the simple model showed that proportions of ploidy levels found in the field would converge quickly to the tetraploid stable state. In other words, diploids, triploids and pentaploids would lose their dominance over two generations and will eventually disappear as the system reaches a stable state with only tetraploids and hexaploids present. Similarly as in the clonal model, tetraploids are increasingly strongly favoured with growing clonality (of all cytotypes) – clonal growth will conserve all cytotypes including triploids, which help tetraploids prevail. However, the observed frequency of both tetraploids (20.2%) and hexaploids (0.04%) at the locality Havraníky does not match the stable state predicted by any of our models. One potential explanation lies in temporal dynamics: populations are currently in a transient state and will reach the stable state with prevailing tetraploids in the future. The rest of the narrative may be the same as in the case of pentaploids (see above). A dedicated study is needed to resolve this problem.

## Conclusions

Our results show that controlled pair-wise crossing experiments supplemented by model-based predictions of stable population states benefit assessments of natural ploidal diversity. Results of such experiments impose a key information needed for the interpretation of field ploidal patterns. In our particular case, the model projections based on results of our crosses suggest that triploid dominance in wild populations of *Pilosella echioides* can only be reached via a relatively high capacity for clonal growth (and proportionally lower sexual reproduction) in all cytotypes combined with higher clonal growth in triploids. This is the most likely process through which clonality can offset the disadvantage of triploids in sexual reproduction and preclude the minority cytotype exclusion.

The system under study also demonstrates that significant numbers of triploids can persist in fully sexual populations; their potential role for structuring ploidal diversity should thus be taken seriously and examined with care. Because of their inherent instability, triploids can lead to formation of higher-ploidy levels (up to hexaploid) and are involved in gene transfer across these ploidy levels. All these phenomena that do not occur in even-ploidy systems and may trigger important evolutionary transitions. Although the role of triploids is suggested by theoretical models [27], this is most likely not the case in the majority of real-world mixed-ploidy populations, where minority (secondary, usually odd-ploidy) cytotypes (if they occur at all) are usually extremely rare and likely originate as a by-product of the coexistence of the main cytotypes. Fertile triploids are currently known only from a handful of sexual taxa (*Chamerion angustifolium*, *Galax urceolata*, *Pilosella echioides*). However, they might have occurred in a much wider array of taxa during brief but evolutionarily significant moments.

Finally, we propose that our approach combining empirical data from crossing experiments with formal modelling is essential for the identification of processes that drive ploidal diversity in the field. It provides a formal link between different types of empirical findings (e.g. proportions in the field and the crossing experiment) and can translate existing information of the system component into predictions that can be directly compared to field data. In our particular case, such comparison indicated that explanations of the ploidal structure in the field should be sought among processes other than sole sexual reproduction. We propose that such approaches may become more common as they are in other fields of population genetics.
